# FeS/FeS_2_ nanoscale structures synthesized in one step from Fe(ll) dithiocarbamate complexes as a single source precursor

**DOI:** 10.3389/fchem.2022.1035594

**Published:** 2022-12-02

**Authors:** Mojeed A. Agoro, Edson L. Meyer

**Affiliations:** ^1^ Fort Hare Institute of Technology, University of Fort Hare, Alice, South Africa; ^2^ Department of Chemistry, University of Fort Hare, Alice, South Africa

**Keywords:** morphology, thermal stability, particle size, optical properties, X-ray diffraction

## Abstract

Nanoscale FeS and FeS_2_ mixed phases were synthesized by one-pot decomposition of (*N*-anil-*N*-piperldtc)Fe1 as FeS#1), (*N*-piperldtc)Fe2 as FeS#2) and (*N*-anildtc)Fe3 as FeS#3) complexes as precursors, with the help of tri-n-octylphosphine oxide (TOPO) coordinating solvent. Their morphology, stability, size, optical and structural characteristics were observed using various material characterization instruments. In comparison to the FeS#2 nano-flower shape, FeS#1 and FeS#3 have a uniform nano-rod shape. A one-step decomposition pattern was obtained from the thermal gravimetric analysis (TGA) results with 3% final mass residual. The high-resolution transmission electron microscopy (HRTEM) image reveals an aggregation and size diameter of around 14.47–30.25 nm for the three samples. The optical response between 3.8 and 4.2 eV from the three samples shows that they are inconsiderable materials for solar cells application. The diffraction peaks for the three samples matched well with the FeS/FeS_2_. These nanoscale materials can be used in a variety of applications, including lithium-ion batteries, biosensors, hydrogen evolution, and multifunctional nanocomposite materials.

## 1 Introduction

In recent years, the replacement of the third generation photovoltaic cells with the ideal dye-sensitized solar cells (DSSCs) with the development of new types with similar structures, such as QDSCs, has gained more ground among many researchers. The replacement of dyes with the introduction of the quantum dot (QD) has proven to be the major difference as a result of its photo stability, tunable band gap and multiple exciton effect (Karki et al., 2015; Zheng et al., 2015; Agoro et al., 2022; Agoro et al., 2021; Agoro et al., 2021). Because of their quantum confinement effects and broad surface-to-volume ratio, metallic nanoparticles have demonstrated tremendously unique chemical, physical, and biological properties (Shi et al., 2006; Pal et al., 2011; Paca, and Ajibade, 2018; Meyer et al., 2019). These account for their adoption in many applications in energy storage, transmission, data storage, communications, environmental protection, optic sensing, biology, cosmetics, and medicine (Shi et al., 2006; Pal et al., 2011; Paca, and Ajibade, 2018; Meyer et al., 2019). Considerable ground has been covered on the synthetic pathways for the formation of nanoparticles. The main challenge remains obtaining nanoparticles with no or low impurities (Khan et al., 2019; O'Brien et al., 2005; Revaprasadu and Mlondo, 2006). Single source precursors (SSPs) have the distinguishing advantage of an easy and cheap preparation route to obtain air and moisture stable nanoparticles using readily available starting materials (Agoro et al., 2020; Agoro et al., 2020; Meyer et al., 2020). O'Brien and co-workers have reported on a number of studies on SSPs, particularly iron sulfides, due to their attractive ability to tune iron to sulfur ratios (Vanitha and O’Brien, 2008; Akhtar et al., 2011; Abdelhady et al., 2012). However, obtaining a phase pure FeS materials is difficult. To avoid stoichiometric variation with undesirable end products, Revaprasadu and colleagues (Khan et al., 2019) consider purpose design requirements to be considered for the SSPs method in the formation of pure phase FeS nanoparticles, such as purity. Non-toxic or low-toxicity reagents should be used; easy stability of the complexes; a simple synthetic step; etc. Irrespective of all this consideration, SSPs still have an edge in producing materials with fewer defects and improved composition through the pre-existing bond between a metal and a chalcogen atom.

Transition metal chalcogenides have proved to be vital compounds for the formation of novel properties useful for many applications (Dutta et al., 2012). Among these materials, iron chalcogenides have gained an upper edge due to their structural, magnetic and semiconducting properties (Dutta et al., 2012). Iron sulfides (FeS) exit in different phases such as troilite, pyrrhotite, mackinawite, marcasite greigite, and pyrite phases. FeS is mostly obtained from dithiocarbamate complexes of Fe (II) species (Roffey et al., 2019). Gao et al. (2008) reported the first iron dithiocarbamate complexes using Fe (II) [Fe(S_2_-CNEt_2_)_2_ (1,10-phen)] and Fe(III) [Fe(S_2_CNEt_2_)_3_] complexes, as well as the effects of solvent and decomposition temperature on troilite (hexagonal FeS) and pyrrhotite (monoclinic) end products. On the other hand, thermolysis of [Fe(S_2_CNEt_2_)_3_] below 300°C with the aid of an oleylamine coordinating solvent formed a mixed phase of greigite and pyrrhotite, while a 320°C increase in temperature resulted in pure pyrrhotite nanosheets (Roffey et al., 2019). O'Brien recently reported the detailed effects of solvent, temperature, and ligand constituents on [Fe(S_2_CNR_2_)_3_] SSPs (Akhtar et al., 2011). These methods produce nanomaterials that are pure and volatile without impurities, with the aid of precursors with good thermal stability to the desired product (Karki et al., 2013; Zheng et al., 2015; Agoro et al., 2022). Furthermore, SSP is used to prepare semiconductor nanoparticles of monodispersed crystalline by replacing poisonous organometallic constituents (Agoro et al., 2020; Agoro et al., 2020; Meyer et al., 2020). This is achieved in an inert environment through coordinating solvents with high boiling points such as capping agents such as hexadecylamine (HDA), trioctylphosphineoxide (TOPO), oleylamine, or octadecylamine (ODA) (Matthews et al., 2016; Buchmaier et al., 2017; Paca. and Ajibade, 2017; Mbese et al., 2019; Mbese et al., 2020; Agoro et al., 2022). These coordinating solvents stabilize and passivate the nanoparticles from aggregation, which gives rise to the formation of modified nanostructures with better optical, morphological, and moisture stability, free of impurities and defect by-products (Matthews et al., 2016; Buchmaier et al., 2017; Paca. and Ajibade, 2017; Mbese et al., 2019; Mbese et al., 2020).

Three different *bis*(dithiocarbamato) iron (II) complex molecular precursors for novel nanostructure FeS QDs nanoparticles are described in this study. The FeS QDs properties were studied using Photoluminescence (PL), Ultraviolet-Visible (UV-Vis), high-resolution transmission electron microscopy (HRTEM), Field emission scanning electron microscopy (FESEM), X-ray diffraction (XRD), energy dispersive X-ray spectroscopy (EDS), thermal gravimetric analysis (TGA) and derivative thermogravimetric (DTG), Atomic force microscopy (AFM) and Fourier Transform Infrared Spectroscopy (FTIR).

## 2 Materials and methods

### 2.1 Materials and reagents

**SCHEME 1:**
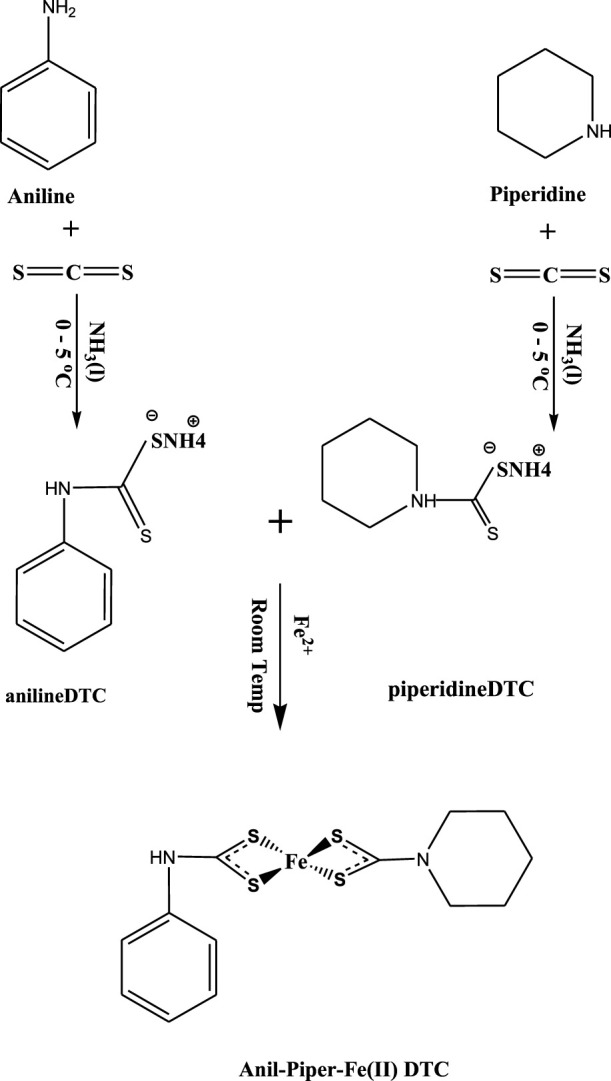
Synthesis of *bis*(*N*-anil-*N*-piperldithiocarbamato)iron (II) complexes.

Ammonium ligands of piperldithiocarbamate and anildithiocarbamate were prepared using modified literature reported by (Agoro et al., 2022) as seen in Scheme 1 and supporting information. Merck (Darmstadt, Germany) provided methanol, iron (II) chloride tetrahydrate salt (FeCl₂*4H₂O), TOPO, and oleic acid (OA), which were used without further purification.

### 2.2 Synthesis of *bis*(*N*-anil-*N*-piperldithiocarbamato)iron (II) complexes as (*N*-anil-*N*-piperldtc)Fe1

At room temperature, (FeCl₂*4H₂O) (2.5 mmol, 0.4971 g) were dissolved in distilled water and mixed with ammonium *N*-piperldithiocarbamate (2.5 mmol, 0.4459 g) and ammonium *N*-anildithiocarbamate (2.5 mmol, 0.4658 g) in a 1:1:1 ratio for 2 h until solid precipitation was obtained. The precipitate was filtered, washed three times with cold methanol, and dried under vacuum as seen in Scheme 1. The same procedure was followed for the two complexes using ammonium *N*-piperldithiocarbamate (2.5 mmol, 0.4459 g) and ammonium *N*-anildithiocarbamate (2.5 mmol, 0.4658 g) at a ratio of (2:1), with three complexes from each ligand formulated as: (*N*-anil-*N*-piperldtc)Fe), (*N*-pierldtc)Fe), and (*N*-anildtc)Fe). (*N*-anil-*N*-piperldtc)Fe) (yield: 72%; M.pt: 222–224°C). ^1^H NMR (DMSO) δ 9.77 (m, 8H-C_6_H_12_), 7.12–7.49 (-C_6_H_12_), 3.31 (s, 2H –NH), 1.24 (t, 2H-CH_2_), 2.5 (s, 1H-SH). ^13^C NMR (DMSO) δ40 (-NH_2_), 40 (-S-C), 130.4 (-C_6_H_12_), 124.1 (-C_6_H_12_), 207 (-CS_2_). Selected IR (cm^−1^) 1484 *v* (C-N), 1208 *v* (C-S), 3220 *v* (N-H), 544 *v* (M-S). UV–Vis (CH_3_OH solution, nm): 266. (*N*-piperldtc)Fe) (yield: 68%; M.pt: 223–225°C). ^1^H NMR (DMSO) δ 6.52–7.33 (m, 8H-C_6_H_12_), 3.33–3.75 (s, 2H −NH), 1.2 (t, 2H-CH_2_), 2.5 (s, 1H-SH). ^13^C NMR (DMSO) δ 40 (-NH_2_), 40 (-S-C), 124.1 (-C_6_H_12_), 205 (-CS_2_). Selected IR (cm^−1^) 1502 *v* (C-N), 1232 *v* (C-S), 3119 *v* (N-H), 522 *v* (M-S). UV–Vis (CH_3_OH solution, nm): 243. (*N*-anildtc)Fe) (yield: 74%; M.pt: 222–224°C). ^1^H NMR (DMSO) δ 9.77 (m, 8H-C_6_H_5_), 3.32 (s, 2H −NH), 1.24 (t, 2H-CH_2_), 2.51 (s, 1H-SH). ^13^C NMR (DMSO) δ40 (-NH_2_), 40 (-S-C), 128.9 (-C_6_H_5_), 207 (-CS_2_). Selected IR (cm^−1^) 1587 *v* (C-N), 1015 *v* (C-S), 3209 *v* (N-H), 682 *v* (M-S). UV–Vis (CH_3_OH solution, nm): 264.

### 2.3 Preparation of iron sulfide nanoparticles

**SCHEME 2:**
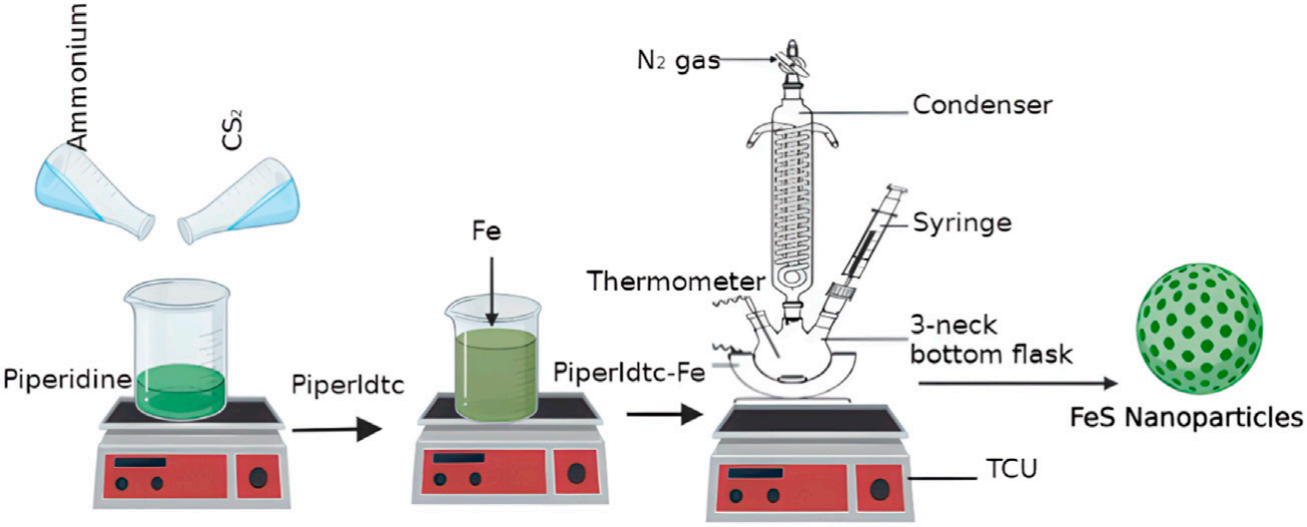
Synthesis of iron sulfide nanoparticles as FeS#1, FeS#2 and FeS#3.

A three-necked flask containing 3 g of TOPO equipped with a thermometer, reflux condenser, and a rubber septum was thermalized to 200°C in an inert environment with vigorous stirring as seen in Scheme 2. The precursor (0.3 g) dissolved in 4 ml of oleic acid was injected into the hot solution with the aid of a syringe. A decrease in temperature of about 15–30°C was observed. For 1 h, the reaction was kept at 200°C. The product in the flask was collected and cooled at 70°C. 20 ml of cold methanol was added, and the synthesized nanomaterials were isolated by centrifugation at 2000 rpm for 30 min. The nanomaterials were washed three times with 30 ml of cold methanol to remove any excess oleic acid and TOPO before drying under vacuum. FeS#1, FeS#2, and FeS#3 nanomaterials were synthesized from (*N*-anil-*N*-piperldtc) Fe1), (*N*-piperldtc) Fe2), and (*N*-anildtc) Fe3), respectively.

### 2.4 Materials characterizations

The optical properties, surface morphology, thermal stability, elemental compositions, and thickness of the FeS nanoparticles were elevated through the following techniques: FE-SEM (S-4200, Hitachi) coupled with EDS on-system, operating at a voltage of 15 kV. The size distributions of the three samples of the three samples were identified using JEOL JEM 2100 HRTEM operating at 200 kV. Their structural patterns were established using XRD analysis by Cu Ka radiation run at 40 mA and 40 kV. Surface roughness was obtained using AFM (JPK NanoWizard II AFM, JPK instruments) at a scan rate of 0.8 Hz. TGA was performed at temperatures ranging from 30 to 600°C at a rate of 10°C min^−1^. FTIR analysis was achieved through the aid of Bruker Platinum ATR model Alpha. The PerkinElmer instrument of the model LAMBDA 365 and LS 45 fluorimeter were used to understand the optical properties (UV-Vis and PL analysis) of the three samples. NMR analysis was carried out using a Bruker AV-400 spectrometer working at 400.13 MHz, 300 K, and a spinning rate of 4 kHz.

## 3 Results and discussions

### 3.1 Thermal gravimetric analysis

**FIGURE 1:**
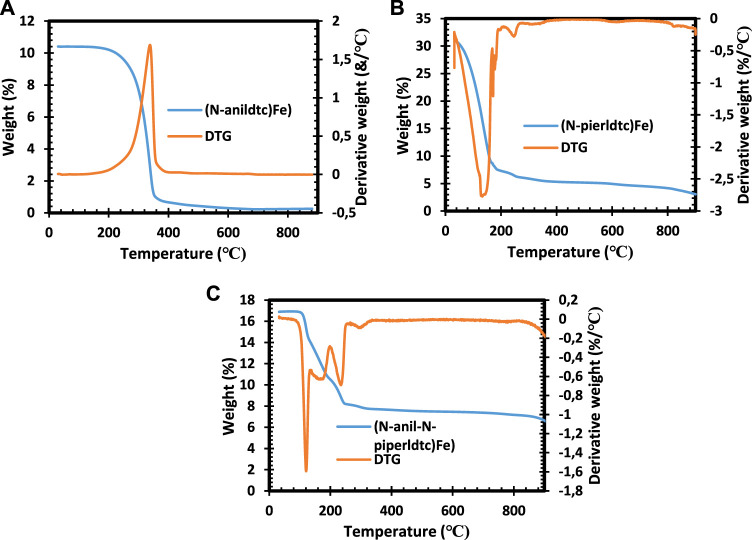
TGA and DTG spectra of (*N*-anil-*N*-piperldtc)Fe1), (*N*-piperldtc)Fe2), and (*N*-anildtc)Fe3) complexes.

Thermogravimetric results for (*N*-anil-*N*-piperldtc) Fe1), (*N*-piperldtc) Fe2), and (*N*-anildtc) Fe3) complexes shown in Figures 1A–C show a one-step decomposition pattern with complete loss at 357°C and a 0.3% final residual, which is less than the calculated values of 8.4%. TGA curves for (*N*-piperldtc) Fe2 and (*N*-anildtc) Fe3 complexes show a two-step decomposition with a significant loss at 274°C and 325°C, respectively (Figures 1B,C). The remaining mass residual of 3% for (*N*-piperldtc)Fe2) is lower compared to the calculated value of 8.2%, while the (*N*-anildtc)Fe3) complexes with a final residual of 7.5% is very close to the calculated value of 8.2% (Akhtar et al., 2014; Mlowe et al., 2016).

### 3.2 X-ray diffraction

**FIGURE 2:**
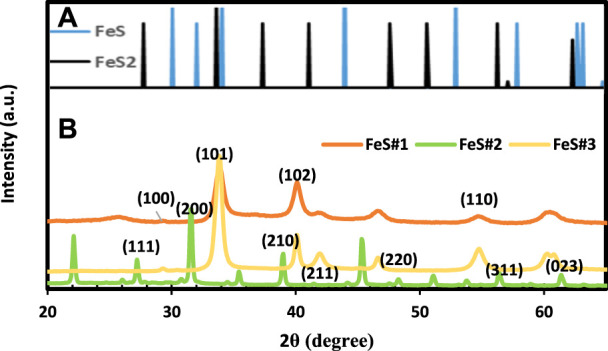
XRD spectra of **(A)** FeS (PDF#65-9124), FeS_1.96_ (ICDD No 01-073-8127) and **(B)** Fes#1, FeS#2 and FeS#3 nanoparticles.

Figure 2 reveals the structural identity of the iron sulfide for the three samples with the aid of X-ray diffraction (XRD) patterns. The FeS#2 diffraction peaks matched well with the standard data (JCPDS42-1340). The major peaks observed for FeS#2 at 27.2°, 31.5°, 39.8°, 45.4°, 48.4°, and 56.9° correspond to the catalog of (111), (200), (210), (211), (220), and (311) planes of the crystalline nature of FeS_2_ phase (see Figure 2). The FeS#1 and FeS#3 nanostructures fabricated from the SSP approach reveal principal peaks from 33.9–55.1⁰ which can be indexed to hexagonal FeS (JCPDS Card No. 03-065-3356) as seen in Figure 2. The diffraction peaks for both samples remain unchanged, with the dominant peak at 33.9⁰ ascribed to the (101) plane of hexagonal FeS pure phase, indicating the successful synthesis of FeS. The findings of this study are consistent with the literature (Wang et al., 2013; Mutalik et al., 2020). The disparity in the phase purity of FeS#1 and FeS#3 with pure hexagonal FeS phases and the FeS#2 with FeS_2_ phase is due to the amount of the carbon chain in the molecular precursor; increasing the carbon chain results in the FeS_2_ phase, which is in agreement with literature by Trinh et al. (2017).

### 3.3 Fourier transform infrared spectroscopy

**FIGURE 3:**
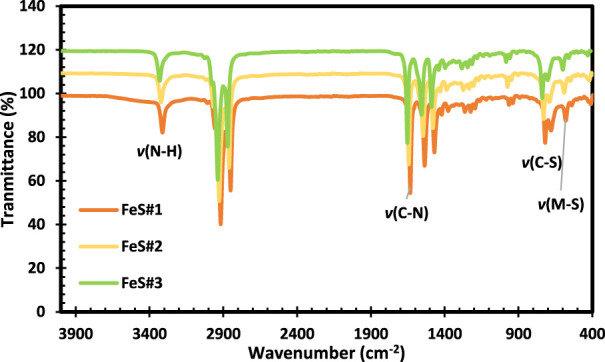
FTIR spectra of Fes#1, FeS#2 and FeS#3 nanoparticles.

The FTIR results for FeS#1, FeS#2, and FeS#3 are shown in Figure 3. The peaks observed at 3310 cm^−1^ from the three FeS correspond to the stretching of the n (N-H) vibrations, confirming the presence of phenyl units in metal sulfides. The broad bands observed around 2916–2852 cm^−1^ for the three samples were assigned to the sp stretching vibrations. The stretching vibrations for C-N were shown to be around 1633, 1539, and 1471 cm^−1^, respectively (Lai and Sreekantan, 2013; Wang et al., 2016). The peaks for C-S and M-S vibration were observed at 719 and 581 cm^−1^ in all the samples. The observed peaks affirmed the presence of all the typical peaks associated with CuS obtained from the molecular precursor of dithiocarbamate complexes as reported by (Paca and Ajibade, 2017; Paca and Ajibade, 2018).

### 3.4 UV-Vis, tauc plots and photoluminescence

**FIGURE 4:**
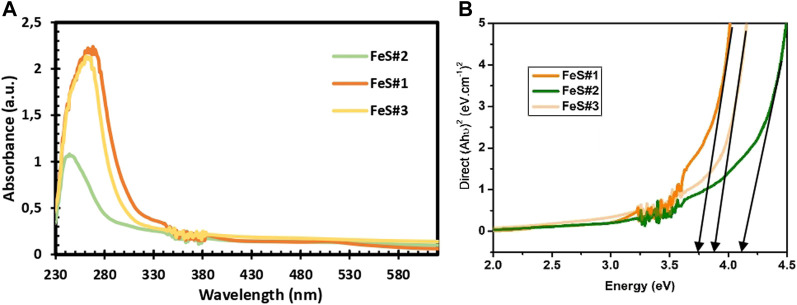
UV-Vis spectra and Tauc plot of Fes#1, FeS#2 and FeS#3 nanoparticles.

UV–Vis spectroscopy in Figure 4A provides the absorption properties of the prepared FeS#1, FeS#2, and FeS#3 nanoparticles. The fundamental absorption edge of the three prepared materials was calculated from the absorption spectra of the three samples. FeS#1 reveals an estimated absorption band gap of 3.8 eV, obtained from Tauc plots (inset, Figure 4B). The FeS#2 and FeS#3 display band gap properties of 4.2 and 3.9 eV. The optical response from the three samples implies that the nanoparticles are structural and size-dependent (Cabán-Acevedo et al., 2014; Rashid et al., 2018). PL analysis is a suitable method to determine the presence of foreign elements and the crystalline quality of a materials. The emission properties of the prepared materials were investigated at room temperature with an excitation wavelength of 350 nm. The emission spectra of FeS#1, FeS#2 and FeS#3 are displayed in Supplementary Figure S1. The highest emissions are found at 484 nm and 512 nm in the blue and green shifts for all the samples. The origin of the emission peaks is related to defects and the band edge emission, i.e., radiative decay from the conduction band to the valence band (Paca and Ajibade, 2017). The prepared FeS#1 from the mixed ligands did not show any superiority in crystalline quality to the FeS#2 and FeS#3 from the single ligands. A summary of the three samples is found in Table 1.

**TABLE 1:** Application of iron metal sulfide nanoparticles prepared through different route with their applications. RT = room temperature.

Methods	Phase	Size (nm)	T.°C	Time	Appearances	Ref.
SSP	FeS	14.47–30.25	200	1 h	Nano-rods	Present study
	FeS_2_	17.78–23.11			Flower-like	
	FeS	17.39–21.74			Nano-rods	
Ball-milling	FeS_2_ and FeS	Microsized	155	6 h	Nanoparticles	Xi et al. (2019)
Facile	FeS	10–20	600	12 h	Sheet-like	Guo et al. (2017)
Hydrothermal	FeS	3.000	180	12 h	Pomegranate flower-like	Jin et al. (2017)
Co-precipitation	FeS	80–102	RT	30 min	Nanoparticles	Agnihotri et al. (2020)
Biomineralization	FeS_2_	7	RT		Nano-dots	Jin et al. (2018)
Hydrothermal	Fe_3_S_4_	2000–5000	220	24 h	Plates	Fu et al. (2019)
Hydrothermal	Fe_3_S_4_	17.7	180	6-24 h	Dispersible nanoparticles	Moore et al. (2019)
Co-precipitation	Fe_3_S_4_	10–20	20–100	10-30 min	Platelet-like	Simeonidis et al. (2016)
High temperature chemical synthesis	Fe_7_S_8_	5,000,500 (thickness)	400	4 h	Nano-sheets	Lai and Sreekantan, (2013)
Co-precipitation	Fe_3_O_4_	15–20	50	24 h	Sponge-like	Alaghmandfard and Madaah Hosseini, (2021)
hydrothermal and annealing	FeS_2_	25	500	1 h	Spherical	Mutalik et al. (2020)
AACVD and precursors	Fe_0.975_S, Fe_0.975_S and FeS_2_	400–450	350	2 h	Sheet-like	Mlowe et al. (2016)
	Fe_0.95_S_1.05_	530–580	400	2 h	Nano-leaf/flake	
		600–650	450	2 h	Nano-leaf/flake	
SSP	Fe_7_S_8_	23.90–38.89	180	1 h	Spherical	Paca and Ajibade, (2018)
	Fe_9_S_10_	4.50–10.60	180	1 h	Spherical	
	FeS	6.05–6.19	180	1 h	Rod-like	

### 3.5 High-resolution transmission electron microscopy

**FIGURE 5:**
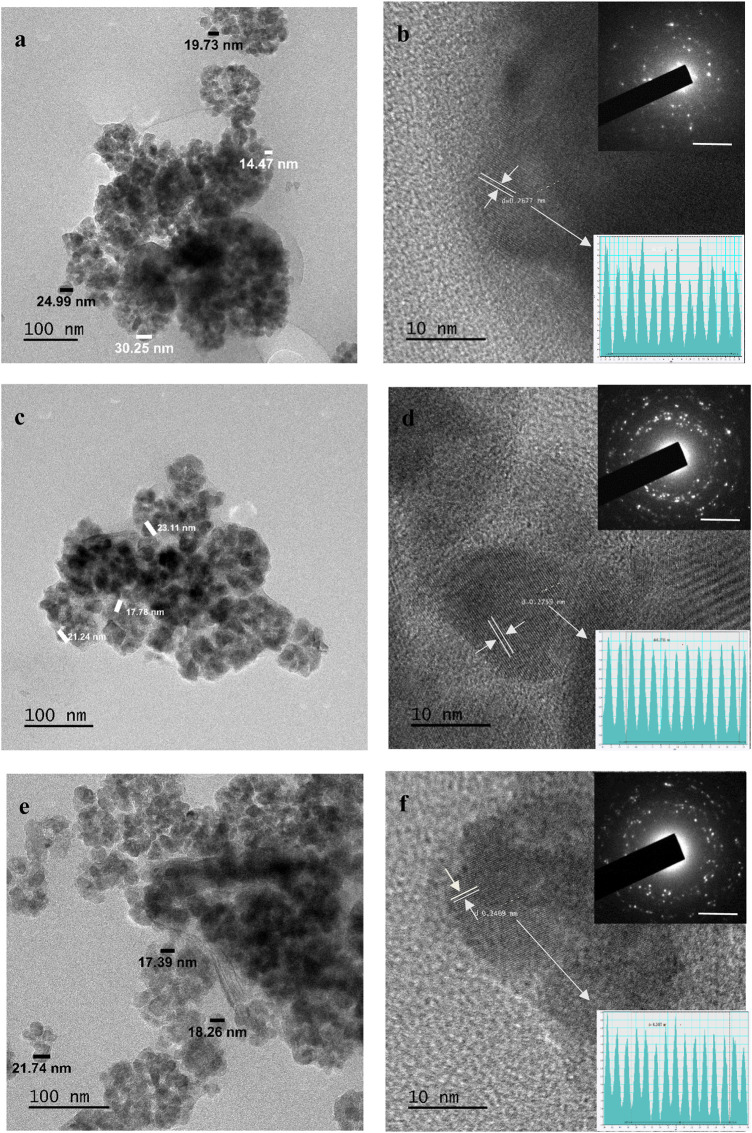
HRTEM images of Fes#1 **(A,B)**, FeS#2 **(C,D)** and FeS#3 **(E,F)** nanoparticles.

The HRTEM images shown in Figures 5A–E illustrate the morphological and structural patterns of iron sulphide nanoparticles for FeS#1, FeS#2, and FeS#3. The HRTEM image presented for FeS#1 as seen in Figure 5A displays an irregular spherical shape with an aggregation and size diameter of around 14.47–30.25 nm. The crystal grains shown from the lattice fringes in Figure 5B for FeS#1 connote polycrystalline nanoparticles with a *d*-spacing of 0.267 nm, corresponding to the (101) hexagonal FeS structure. The bright rings of the SAED patterns in Figure 5C for FeS#1 confirm the crystalline nature, while the diffused rings are linked to the amorphous coordinating solvent (Paca and Ajibade, 2018). The TEM images in Figure 5C illustrate in detail the hierarchical inner structure of the FeS#2 nanoparticles with a great interconnect structure, with a diameter of around 17.78–23.11 nm. HRTEM images and SAED are presented in Figure 5D, which gives a well-resolved crystalline structure with an interplanar distance of 0.275 nm indexed to the (200) plane of FeS (Wang et al., 2016; Xi et al., 2019). FeS#3 reveals an intrinsic crystallogical nanocrystal structure as seen in the HRTEM and the inset SAED images in Figures 5E,F. The particles spanned around 17.39 nm–21.74 nm with various amounts of small crystal grains, revealing the polycrystalline nature of FeS#3, with a dominant lattice *d*-spacing of 0.267, which is linked to the hexagonal FeS structure of (101) planes (Wang et al., 2016). The weight of the molecular precursor complexes accounts for the disparity in the particle size distributions, which is also supported by the literature (Di Giovanni et al., 2014).

### 3.6 Field emission scanning electron microscopy and energy dispersive X-ray spectroscopy

**FIGURE 6:**
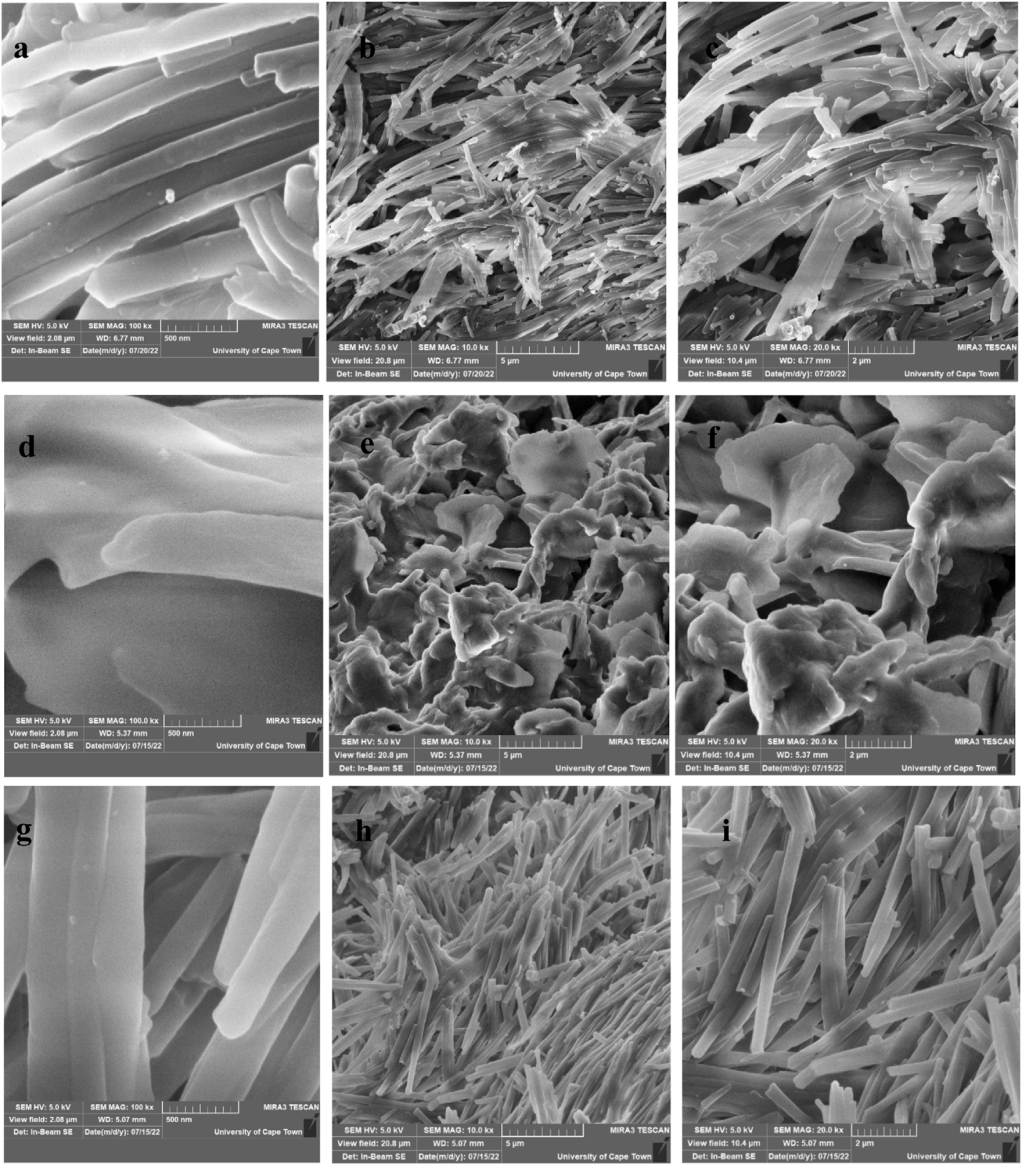
FESEM images of Fes#1 **(A–C)**, FeS#2 **(D–F)** and FeS#3 **(G–I)** nanoparticles.

**FIGURE 7:**
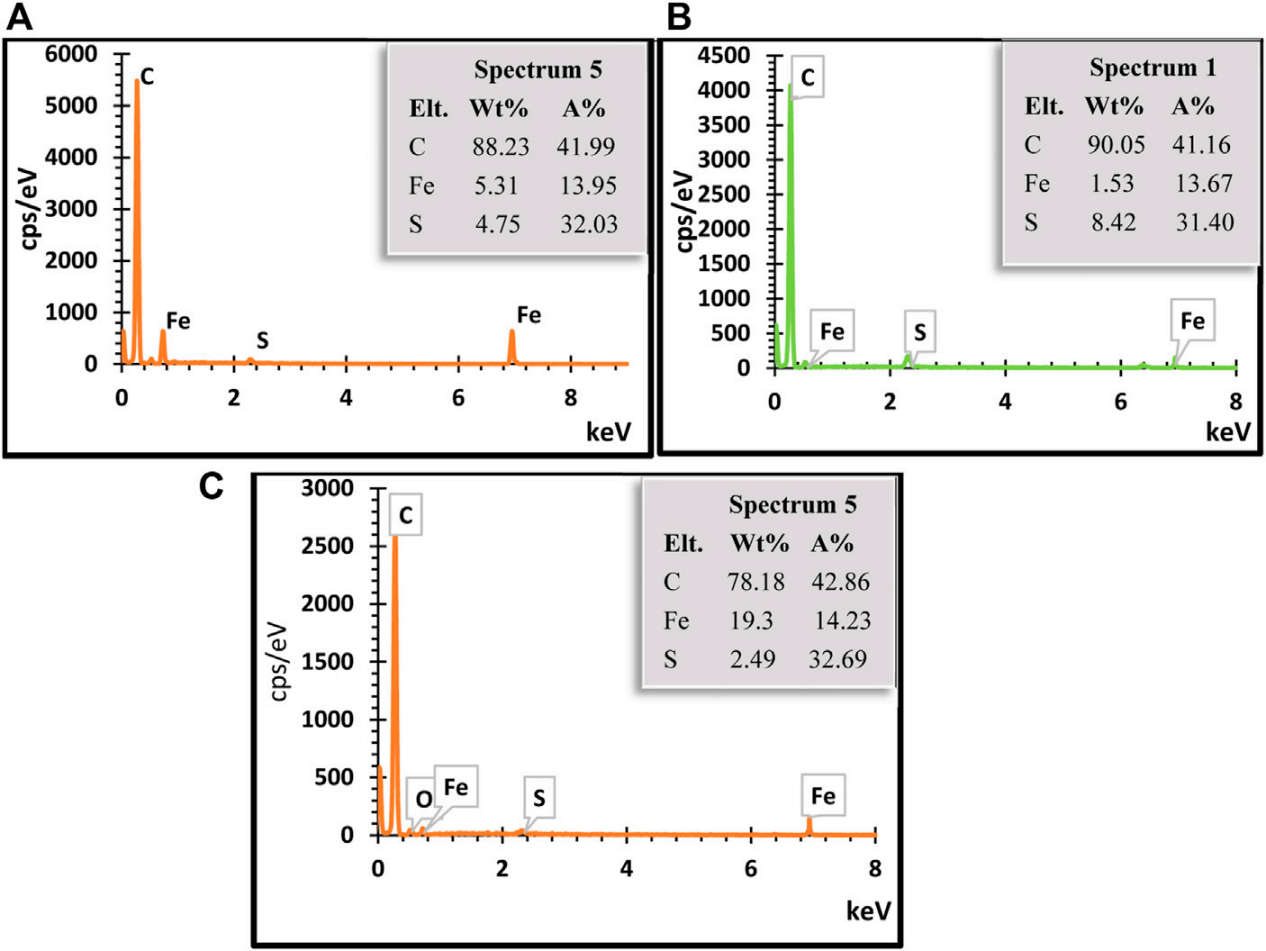
EDS spectra of **(A)** Fes#1, **(B)** FeS#2 and **(C)** FeS#3 nanoparticles.

FESEM micrographs from various magnification images of the prepared iron sulfide nano-rods as illustrated in Figures 6A–I. The micrographs of both FeS1 and FeS#3 nano-rods shown in Figures 6A–C and g-i reveal striped surfaces, sharp structured heads, and growth directions that are conically and vertically shaped. By further increasing the magnification, their large crystal agglomerates consist of layers on each other with uniformly shaped nano-rods for both samples, following the pattern of the XRD results. Low and high magnification FESEM images of FeS#2 (Figures 7D–F) show a nano-sheet shape that is stacked together to form nano-flowers (Li et al., 2020). Apart from the difference in the working principle and text condition of SEM and TEM. Coz et al., 2008 reveal that difference in TEM and SEM properties such as morphology, size distribution, mixing state, and chemical composition vary dramatically as a function of space, time and sulfur content of the samples. This further cement the change in weight percentage of sulfur content in the EDS spectrum. EDS elemental weight percentage analysis of the three samples indicates the presence of C, O, Fe, and S all exist as seen in Figures 7A–C (Guo et al., 2017).

## 4 Conclusion

Decomposition of dithiocarbamate Fe (ll) complexes as precursors was used to prepare nanoscale in this paper using the single source precursor method. The high emissions in the blue and green shift for all the samples are related to defects and the band edge emission. This shows that the mixed ligand complexes do not have superiority over the single ligand complexes. The weight percentage of the three samples confirms the purity of nature with a high concentration of Fe and S. The size-dependent and structural properties imply that the energy band gap of around 3.8–4.2 eV is an indication that the prepared materials will only absorb a very small portion of the solar spectrum, making them an inconsiderable candidate for applications such as QDSSCs. However, the prepared, cost-effective nanoparticles could be explored in other applications like hydrogen evolution, energy storage devices, biosensors, etc.

## Data Availability

The original contributions presented in the study are included in the article/Supplementary Material; further inquiries can be directed to the corresponding author.
