# Supplementary material for: FeS/FeS2 nanoscale structures synthesized in one step from Fe(ll) dithiocarbamate complexes as a single source precursor
Source: Front Chem. 2022 Dec 2;10:1035594. doi: 10.3389/fchem.2022.1035594 (PMC9755493; doi:10.3389/fchem.2022.1035594)
Supplement: Supplementary file 1 [file DataSheet1.pdf]

Supporting Information

FeS/FeS_2 nanoscale structures synthesized in one step from Fe(ll) dithiocarbamate complexes as a single source precursor

Mojeed A. Agoro[1, 2]*and Edson L. Meyer[1]

[1] Fort Hare Institute of Technology, University of Fort Hare, Private Bag X1314, Alice 5700, Eastern Cape, South Africa.; magoro@ufh.ac.za (M.A.A); emeyer@ufh.ac.za (E.L.M)
[2] Department of Chemistry, University of Fort Hare, Private Bag X1314, Alice 5700, Eastern Cape, South Africa; magoro@ufh.ac.za (M.A.A)
* Correspondence: magoro@ufh.ac.za, amodoyin@gmail.com (M.A.A) +27781246437)

Characterization of piperldithiocarbamate and anildithiocarbamate

[Piperldtc)]: Yield: 10.6962 g, (85.70%), M.pt. 196-198 C; ^{1}H NMR (DMSO) δ 6.5-7.3 (m, 8H-C_6H_{12}), 3.3 (s, 2H –NH), 1.24 (t, 2H-CH_2), 2.51 (s, 1H-SH). ^{13}C NMR (DMSO) δ 40 (-NH_2), 40 (-S-C), 132.7 (-C_6H_{12}), 205 (-CS_2). Selected IR (cm^{-1}) 1412 *v*(C-N), 1219 *v*(C-S), 3296 *v*(N-H). UV–Vis (CH_3OH solution, nm): 401. **[Anildtc)]:** Yield: 5.4198 g, (77.60%), M.pt. 194-1964 C; ^{1}H NMR (DMSO) δ 7.1-7.49 (m, 8H-C_6H_5), 3.32 (s, 2H –NH), 1.24 (t, 2H-CH_2), 2.51 (s, 1H-SH). ^{13}C NMR (DMSO) δ 40 (-NH_2), 40 (-S-C)124.1-128.9 , (-C_6H_5), 207 (-CS_2). Selected IR (cm^{-1}) 1412 *v*(C-N), 1219 *v*(C-S), 3419 *v*(N-H). UV–Vis (CH_3OH solution, nm): 315.

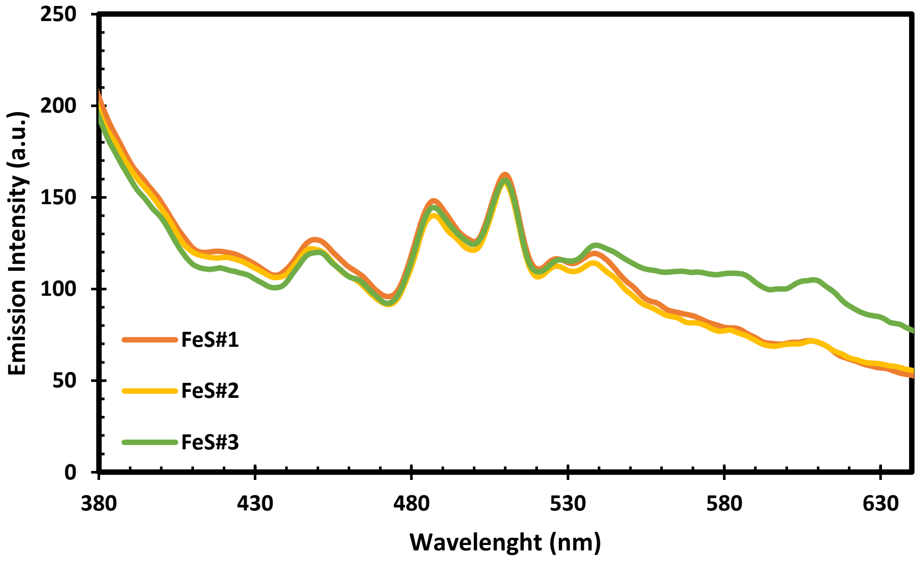

Figure S1: PL spectra of Fes#1, FeS#2 and FeS#3 nanoparticles.
